# Outcomes of Bedaquiline Treatment in Patients with Multidrug-Resistant Tuberculosis

**DOI:** 10.3201/eid2505.181823

**Published:** 2019-05

**Authors:** Lawrence Mbuagbaw, Lorenzo Guglielmetti, Catherine Hewison, Nyasha Bakare, Mathieu Bastard, Eric Caumes, Mathilde Fréchet-Jachym, Jérôme Robert, Nicolas Veziris, Naira Khachatryan, Tinatin Kotrikadze, Armen Hayrapetyan, Zaza Avaliani, Holger J. Schünemann, Christian Lienhardt

**Affiliations:** St. Joseph’s Healthcare Hamilton, Hamilton, Ontario, Canada (L. Mbuagbaw);; Centre for the Development of Best Practices in Health, Yaoundé, Cameroon (L. Mbuagbaw);; McMaster University, Hamilton (L. Mbuagbaw, H.J. Schünemann);; Centre d'Immunologie et des Maladies Infectieuses, INSERM, Paris (L. Guglielmetti, E. Caumes, J. Robert, N. Veziris);; Centre Hospitalier de Bligny, Bris-sous-Forges, France (L. Guglielmetti, M. Fréchet-Jachym);; Sorbonne Université, Paris, France (L. Guglielmetti, J. Robert, N. Veziris);; Médecins Sans Frontières, Paris (C. Hewison);; Janssen Research & Development, LLC, Titusville, New Jersey, USA (N. Bakare); Epicentre, Paris (M. Bastard);; Hôpitaux Universitaires de l'Est Parisien, Paris (N. Veziris);; Médecins Sans Frontières, Yerevan, Armenia (N. Khachatryan);; Médecins Sans Frontières, Tbilisi, Georgia (T. Kotrikadze);; National Tuberculosis Control Centre, Yerevan (A. Hayrapetyan);; National Centre for Tuberculosis and Lung Disease, Tbilisi (Z. Avaliani);; World Health Organization, Geneva, Switzerland (C. Lienhardt);; Université de Montpellier, Montpellier, France (C. Lienhardt)

**Keywords:** multidrug resistant, extensively drug resistant, tuberculosis, bedaquiline, individual patient data, tuberculosis and other mycobacteria, antimicrobial resistance, France, Georgia, Armenia, South Africa, China, Europe, Asia, bacteria, XDR, MDR, TB

## Abstract

Bedaquiline is recommended by the World Health Organization for the treatment of multidrug-resistant (MDR) and extensively drug-resistant (XDR) tuberculosis (TB). We pooled data from 5 cohorts of patients treated with bedaquiline in France, Georgia, Armenia, and South Africa and in a multicountry study. The rate of culture conversion to negative at 6 months (by the end of 6 months of treatment) was 78% (95% CI 73.5%–81.9%), and the treatment success rate was 65.8% (95% CI 59.9%–71.3%). Death rate was 11.7% (95% CI 7.0%–19.1%). Up to 91.1% (95% CI 82.2%–95.8%) of the patients experienced >1 adverse event, and 11.2% (95% CI 5.0%–23.2%) experienced a serious adverse event. Lung cavitations were consistently associated with unfavorable outcomes. The use of bedaquiline in MDR and XDR TB treatment regimens appears to be effective and safe across different settings, although the certainty of evidence was assessed as very low.

In 2017, there were ≈10 million (range, 9.0–11.1 million) new cases of tuberculosis (TB) worldwide, of which ≈558,000 were rifampin-resistant TB (RR TB) or multidrug-resistant TB (MDR TB) (*1*). MDR TB refers to resistance to isoniazid and rifampin, 2 of the most powerful TB drugs, with or without resistance to other first-line drugs. Extensively drug-resistant tuberculosis (XDR TB), a more severe form of drug-resistant TB, is defined as MDR TB with additional resistance to any fluoroquinolone and to any of the 3 second-line injectables (amikacin, capreomycin, or kanamycin) (*2*). Treatment outcomes in patients with MDR TB are generally poor, with treatment success in about half of those who receive treatment (56.4%), and much worse in patients with XDR TB (*3*). In 2017, MDR TB and RR TB caused ≈230,000 deaths (*1*). The treatment of MDR and XDR TB is complex and expensive, requiring the use of >4 medications considered to be active in longer regimens (18–20 months) (*4**–**6*), and is fraught with many adverse events that can be debilitating or life threatening (*7**,**8*).

Bedaquiline is a new compound belonging to the diarylquinoline class used to treat MDR TB; cure and culture conversion rates using bedaquiline are promising (*9**,**10*). A recent cost-effectiveness analysis showed that bedaquiline added to a background MDR TB regimen would improve health outcomes and reduce costs in high TB burden countries (*11*). Bedaquiline received accelerated approval in the United States in 2012 for the treatment of pulmonary MDR TB as part of an appropriate combination therapy in adult patients with resistance or intolerability to other treatment regimens. However, in 2013, limited data and concerns about higher death rates among patients who received bedaquiline in the phase II randomized controlled trial (*10*) led the World Health Organization (WHO) to issue an interim conditional recommendation on its use under specific conditions: proper patient inclusion, signed informed consent, adherence to the WHO-recommended principles of designing an MDR TB regimen, close monitoring, and active pharmacovigilance (*12*). Since 2013, many countries have introduced bedaquiline as part of their management strategy for MDR TB, and in 2018, WHO updated its guidance for the use of bedaquiline in MDR TB, including children >6 years of age (*6*). 

In 2016, to update the interim guidance, the WHO Guideline Development Group (GDG) conducted a review of newly available data on the use of bedaquiline in the treatment of MDR TB (*13*). After the publication of the GDG report, further outcome data were retrieved from cohorts in Armenia and Georgia. We report the results of the updated analysis from 5 cohorts of patients with MDR TB treated with bedaquiline, taking into account both study-level and patient-level characteristics on outcomes, including deaths. We also report on the use of bedaquiline in research and nonresearch settings and on adverse events.

## Materials and Methods

### Data Sources

We pooled data from 5 cohorts of patients with MDR or XDR TB treated with bedaquiline as part of compassionate use, programmatic use, expanded access, or research programs. The processes and methods used to search, screen, and select studies have been reported previously (*13*). In brief, studies and datasets were considered only if they met the following inclusion criteria: participants received a diagnosis of MDR TB and were treated with bedaquiline for >6 months as part of an anti-TB regimen. We excluded studies of bedaquiline-only therapy, studies not providing details of the background regimens, studies not providing outcome information, and studies with <10 participants. We contacted national TB programs, nongovernmental organizations, and the drug manufacturer, Janssen Therapeutics (https://www.janssen.com), for unpublished data that fit the criteria. 

Our search retrieved 674 studies, of which only 5 were eligible. The 5 datasets that were finally included for the individual patient data (IPD) meta-analyses originated from Médecins Sans Frontières, which contributed 2 cohorts on behalf of the national TB programs of Armenia and Georgia; the national TB program of South Africa; the Hospital of Bligny, France; and Janssen Therapeutics. All the cohort studies have been published in complete form (*14**–**17*) (Table 1). 

**Table 1:** Characteristics of cohorts in study of bedaquiline treatment for multidrug-resistant tuberculosis

Cohort	Design	Location	Sample size	Inception date	Type of care
Pym 2013 (*14*)	Phase II, single-arm open-label trial	31 sites, 11 countries*	233	2009 Aug	Research
Guglielmetti 2017 (*15*)	Retrospective cohort	France	45	2010 Jan	Expanded access
Ndjeka 2018 (*16*)	Prospective cohort	South Africa	195	2013 Mar	Compassionate use
Hewison 2018 (*17*)	Prospective cohort	Armenia	62	2013 Apr	Compassionate use
	Prospective cohort	Georgia	30		Compassionate use (20), programmatic use (10)

*China, Estonia, Republic of Korea, Latvia, Peru, Philippines, Russian Federation, South Africa, Thailand, Turkey, Ukraine.

### Data Management

We invited the investigators of each study to provide data on the basis of a formal data sharing agreement. We used only anonymized data in this study. We cleaned, recoded, and merged data from the 5 cohorts and saved the data on a secure server at the biostatistics unit of St Joseph’s Healthcare Hamilton/McMaster University (Hamilton, Ontario, Canada). We contacted investigators of each study to ensure accuracy after recoding. We modified categorical variables to match the most commonly used format to ensure consistency across studies. For example, for data from chest radiography, the presence or absence of lung cavitation was the most commonly reported format for findings, so we collapsed data pertaining to the site of the cavitation (left or right lung) or the number of cavitations. We corrected QT intervals for heart rate using the Fridericia formula (QTcF) (*18*). We categorized drug resistance in order of increasing severity as MDR TB (resistance to isoniazid and rifampin, with or without resistance to other first-line drugs), MDR TB + FLQ (additional resistance to fluoroquinolones), MDR TB + INJ (additional resistance to second-line injectables), and XDR (resistance to at least isoniazid and rifampin, and to any fluoroquinolone, and to any second-line injectables). 

### Outcomes

Using standard WHO definitions, we measured the following treatment outcomes: cure, treatment completion, treatment success (the sum of cure and treatment completion), loss to follow-up, and death (*19*). We computed culture conversion at 6 months as 2 consecutive cultures, taken >30 days apart, found to be negative before or at the end of the sixth month of treatment. Adverse event severity and seriousness were defined as by the US Food and Drug Administration (*20*), or as reported by investigators. We measured the following adverse event outcomes: any adverse event, any serious adverse event, number of adverse events by body system, and QT interval prolongation (highest recorded QTcF value and increase from baseline).

### Statistical Methods

We summarized baseline data as mean (+ SD) for continuous variables and frequency (%) for categorical variables. We conducted a random effects meta-analysis of proportions in the first instance to pool effect sizes for effectiveness and safety. The random effects model incorporates the heterogeneity between studies and redistributes the weights of the studies based on this heterogeneity. We assessed statistical heterogeneity using the I^2^ statistic, a measure of heterogeneity between studies. We reported variables and outcomes with various levels of completeness and highlighted them where appropriate. For example, we computed culture conversion only if cultures were examined at the sixth month and not later. Similarly, we used baseline QTcF data only if data were collected within 1 month of starting bedaquiline. We used generalized estimation equations to model the effect of individual- and study-level characteristics on outcomes. We built separate models for the dependent binary (yes/no) variables, culture conversion at 6 months, treatment success, and death, using an unstructured correlation matrix and the logit link. The independent variables (age, sex, HIV status, presence of lung cavitations, severity of drug resistance, and previous use of second-line drugs) are all known to affect outcomes in TB (*21**,**22*). We assessed model fit using the quasi-likelihood under independence model criterion and set the level of statistical significance at α = 0.05. We used different numbers of patients for each analysis because of variations in completeness and availability of data. Samples used for each analysis are shown in Appendix Table 1.

### Certainty Evaluation

We assessed the certainty of the evidence using the Grading of Recommendations, Assessment, Development, and Evaluation (GRADE) approach, which categorizes each outcome by how confident we are that the effect estimate is close to the quantity of interest (*23*). Using this approach, the certainty rating across studies can be high, moderate, low, or very low. We summarized the results and certainty as evidence profiles.

## Results

We included a total of 537 participants in the data analysis. Baseline characteristics are shown in detail by cohort and overall in Table 2. The mean age was 36.4 years (SD 11.8). Two thirds of the participants were men (342; 63.7%); 138 (25.7%) were HIV positive; 341 (99.7%) had pulmonary TB, 253 (73.9%) with lung cavities; and 188 (35.0%) had XDR TB. The key differences between the datasets were a higher proportion of male participants in the cohorts from France and Armenia; 63.1% participants having concurrent HIV in the South Africa cohort; and complete outcome data being available from only 51% of all participants because others were still receiving treatment at the time we collected data. Of note, 36 (6.7%) patients received bedaquiline for >6 months.

**Table 2:** Baseline characteristics of participants in study of bedaquiline treatment for multidrug-resistant tuberculosis*

Variable	Cohort					Total, n = 537
	South Africa, n = 195	France, n = 45	Janssen, n = 205	Armenia, n = 62	Georgia, n = 30	
Mean age, y (SD)	35.8 (11.2)	37.4 (12.1)	34.9 (12.2)	41.6 (12.6)	38.7 (11.9)	36.4 (11.8)
Sex, no. (%)						
M	98 (50.3)	36 (80.0)	132 (64.4)	55 (88.7)	21 (70.0)	342 (63.7)
F	97 (49.7)	9 (20.0)	73 (35.6)	7 (11.3)	9 (30.0)	195 (36.3)
Mean no. months on BDQ (SD)	5.8 (1.2)	12.3 (7.0)	5.9 (1.1)	5.6 (1.6)	6.0 (1.3)	6.37 (2.3)
No. on BDQ >6 mo (%)	4 (2.1)	32 (71.1)	0.0	0.0	0.0	36 (6.7)†
Mean total treatment duration, mo (SD)	14.9 (6.7)	19.4 (4.7)	21.8 (7.6)	20.2(7.4)	14.0 (6.1)	18.47 (6.9)†
No. (%) with treatment outcome available	101 (51.8)†	45 (100.0)	205 (100.0)	62 (100.0)	30 (100.0)	443 (82.5)
No. (%) HIV positive‡	120 (63.1)	2 (4.4)	8 (4.0)	4 (6.5)	1 (3.3)	135 (25.1)
No. (%) on antiretroviral therapy	110 (56.4)	2 (4.4)	0.0	0	0	112 (20.9)
Type of TB, no. (%)						
Pulmonary	NR	44 (97.8)	205 (100.0)	62 (100.0)	30 (100.0)	341 (99.7)
Extrapulmonary	NR	8 (17.8)	0	0	0	8 (2.3)
No. (%) with previous TB treatment	NR	34 (75.6)	193 (94.1)	62 (100.0)	29 (96.7)	271 (79.2)
No. (%) with previous second-line TB treatment	NR	27 (60.0)	177 (86.3)	62 (100.0)	29 (96.7)	295 (86.3)
No. (%) with lung cavities on chest radiograph	NR	39 (86.7)	135 (65.8)	55 (88.7)	24 (80.0)	253 (73.9)
Resistance profile, no. (%)§						
MDR TB	0	7 (15.6)	93 (45.4)	6 (9.7)	0	100 (18.6)
MDR TB + FQ	73 (37.4)	8 (17.8)	31 (15.1)	26 (41.9)	5 (16.7)	147(27.3)
MDR TB + INJ	29 (14.9)	6 (13.3)	13 (6.3)	7 (11.3)	0	55 (10.2)
XDR TB	77 (39.5)	24 (53.3)	37 (18.0)	23 (37.1)	25 (83.3)	188 (35.0)

*BDQ, bedaquiline; FQ, fluoroquinolone; INJ, injectable; MDR, multidrug resistant; NR, not reported; TB, tuberculosis; XDR, extensively drug resistant.  †Missing data: South Africa = 15. ‡Missing data: South Africa = 17; Janssen (drug manufacturer) = 7.  §Missing data: South Africa = 16; Janssen (drug manufacturer) = 31.

The baseline regimens we used in the cohorts varied according to local treatment guidelines, drug susceptibility results, or both. Lamivudine, nevirapine, efavirenz, and tenofovir were the most frequently used drugs among the patients on antiretroviral therapy (Appendix Tables 2, 3).

We computed culture conversion at 6 months only for patients who had a positive sputum culture at baseline and 2 consecutive culture readings >30 days apart, the last taken at the end of the sixth month. Thirty-seven patients did not have sufficient culture data. Of 406 patients with sufficient culture data, the overall culture conversion rate at 6 months was 78.0% (95% CI 73.5%–81.9%; I^2^ = 46%). A total of 443/537 (82.5%) participants had end-of-treatment outcome data: cure, 60.1% (95% CI 50.2%–69.2%; I^2^ = 66%); treatment success, 65.8% (95% CI 59.9%–71.3%; I^2^ = 38%); death, 11.7% (95% CI 7.0%–19.1%; I^2^ = 71%); treatment failure, 5.1% (95% CI 1.6%–14.8%; I^2^ = 73%); and loss to follow-up, 14.8% (95% CI 11.6%–18.7%; I^2^ = 7%).

Safety data were available from a total of 565 participants, including an additional 28 participants from the Janssen cohort who were not eligible for efficacy analyses because they were missing a confirmation of MDR (n = 3) or a positive culture at baseline (n = 25). Of these participants, 91.1% (95% CI 82.2%–95.8%) experienced occurrence of any adverse event and 11.2% (5.0%–23.2%) occurrence of any serious adverse event. The most frequent adverse events were gastrointestinal (16.4%; 95% CI 10.8%–22.9%), nervous system (12.7; 95% CI 6.7%–20.2%), and hepatic (8.6%; 95% CI 1.4%–20.7%) (Figure).

**Figure:**
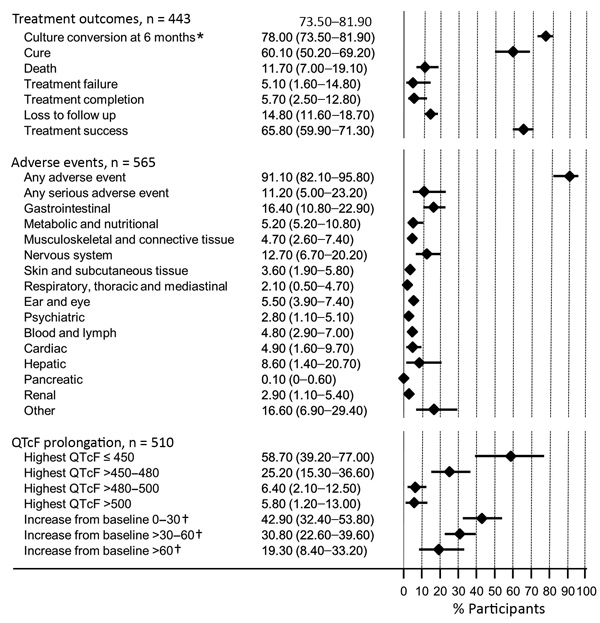
Summary of treatment outcomes and adverse events in study of bedaquiline treatment for multidrug-resistant tuberculosis. Values are shown as percent with 95% CI, shown in the graph as horizontal bars. QTcF indicates QT intervals corrected for heart rate using the Fridericia formula. * A total of 406 study participants with culture data at the 6-month point; †, a total of 509 participants with baseline QTcF data.

We found that 5.8% of 510 participants (95% CI 1.2%–13.0%; I^2^ = 84%) had a highest QTcF >500 ms and 19.3% of 509 (95% CI 8.4%–33.2%; I^2^ = 93%) had an increase in QTcF from baseline of more >60 ms. Despite the small sample of patients receiving bedaquiline for a prolonged period (i.e., >6 months), data seem to indicate an absence of effect of exposure to bedaquiline for >6 months on QTc prolongation >500 ms.

### Adjusted Analyses

Culture conversion at 6 months was less likely in patients with a more severe resistance profile (aOR 0.57, 95% CI 0.43–0.76; p<0.001) and with lung cavitations (aOR 0.30, 95% 0.13–0.70; p = 0.004). Treatment success was less likely in patients with lung cavitations (aOR 0.38, 95% CI 0.21–0.68; p = 0.001) and in those with HIV infection (aOR 0.35, 95% CI 0.12–0.99; p = 0.05). The presence of lung cavitations was associated with death (aOR 5.31, 95% 1.25–22.52; p = 0.023) (Table 3).

**Table 3:** Multivariable analyses for key outcomes in study of bedaquiline treatment for multidrug-resistant tuberculosis

Covariate	Culture conversion at 6 mo, n = 318			Success, n = 325			Death, n = 325	
	Adjusted OR (95% CI)	p value		Adjusted OR (95% CI)	p value		Adjusted OR (95% CI)	p value
Male sex	1.25 (0.65–2.41)	0.499		1.27 (0.74–2.15)	0.382		0.60 (0.24–1.47)	0.264
Age, y	1.01 (0.99–1.04)	0.342		0.99 (0.98–1.01)	0.550		1.05 (1.01–1.09)	0.010
HIV positive	0.42 (0.13–1.39)	0.155		0.35 (0.12–0.99)	0.050		0.97 (0.09–10.05)	0.982
Resistance profile†	0.57 (0.43–0.76)	<0.001		0.84 (0.68 −1.04)	0.110		1.14 (0.73–1.79)	0.562
Presence of lung cavitation	0.30 (0.13–0.70)	0.004		0.38 (0.21–0.68)	0.001		5.31 (1.25–22.52)	0.023
Previous use of second-line drugs	0.67 (0.22–2.01)	0.437		0.73 (0.33–1.59)	0.423		1.22 (0.29–5.15)	0.783

*Absence of data on cavitation precluded use of data from South Africa. MDR, multidrug resistant; TB, tuberculosis; XDR, extensively drug resistant.  †Resistance profiles used in this study were MDR TB (reference), MDR TB plus fluoroquinolone, MDR TB plus injectable drugs, and XDR TB.

The GRADE evidence profile is reported in Appendix Table 4. The GDG judged evidence for all outcomes to be of very low certainty; reasons were the risk for bias (lack of control data), risk for inconsistency (considerable statistical heterogeneity), imprecision (wide confidence intervals), and indirectness (variations in adverse event definition) (*13*).

## Discussion

Inclusion of bedaquiline for >6 months in the treatment regimen was associated with good outcomes in these cohorts, with 78% culture conversion at 6 months and a 65.8% treatment success rate, indicating a favorable efficacy of this medicine. Observed death rate was 11.7%. Although almost all patients experienced at least an adverse event (91.1%), only 11.2% experienced a serious one. Only 5.8% of patients had a highest recorded value of QTcF >500 ms and 19.3% had an increase QT from baseline of more than 60 ms. Being older, having a more severe resistance profile, concurrent HIV, and lung cavitation were associated with unfavorable outcomes.

Such results compare favorably with those observed in large cohorts of patients with MDR TB in the prebedaquiline era (with success rates of 54%–58% and death rates of 13.8%–15%) (*7**,**24*), thus indicating a beneficial effect of the addition of bedaquiline to background MDR/XDR TB regimens. Our findings are in line with those from a large South Africa cohort in which the additional use of bedaquiline reduced the risk for death in patients with MDR TB, compared with standard regimens (*25*). In a matched subset of patients from the South Africa cohort, switching from second-line injectables (because of intolerance) to bedaquiline led to fewer unfavorable outcomes (death, loss to follow-up, or treatment failure) (*26*). Likewise, an individual patient data meta-analysis of 50 studies (including this study’s cohort data from France, South Africa, and Janssen Therapeutics) reported lower odds of death with the use of bedaquiline (*27*). The lower mortality rate we observed strengthens the case for the use of bedaquiline in patients with MDR TB. On the basis of data cumulated since 2012 from various observational and programmatic studies, including the South Africa cohort (*25*), WHO has now consolidated its recommendation for the use of bedaquiline, now proposed as a Group A drug (medicines to be prioritized) in longer MDR TB regimens (*6*).

Very few patients in our study had a highest recorded value of QTcF >500 ms. Other studies have found similarly low rates of cardiotoxicity (*28*). This finding could therefore alleviate some of the concerns around the risk for cardiotoxicity related with the use of bedaquiline (*28*).

Our study adds some information about the use of bedaquiline in persons living with HIV and indicates that favorable outcomes may be more challenging to achieve in these patients, bearing in mind that not all of them were on antiretroviral therapy as recommended by WHO. This information is important for countries with a high prevalence of HIV/TB co-infection. However, other reports indicate that the reductions in number of deaths are similar or better in persons living with HIV (*13**,**25*).

Our findings are similar to those observed in other large multicenter studies in terms of success, death, and adverse events (*4*), although higher culture conversion rates at 6 months were reported from studies in Belarus and Germany (*29**,**30*). Our findings should be interpreted with due consideration of the severity of illness in this cohort, in which up to 35% of patients had XDR TB, 86.3% previous second-line treatment, and 73.9% lung cavitations, suggesting that a success rate of 65.8% is probably higher than would otherwise be expected.

Currently, WHO does not recommend the routine use of bedaquiline for longer than 6 months (*6*). However, WHO acknowledges that “clinicians and national TB programs may be compelled to use [bedaquiline] beyond 24 weeks in selected MDR TB patients (including those with additional drug resistance), if the regimen is unlikely to achieve cure or poses a risk [of] creating additional drug resistance” (*31*). We were unable to properly investigate the effect of prolonged bedaquiline use in this study, given the small number of patients (6.7%) who received the medicine for >6 months and the risk for bias by indication (bedaquiline offered to patients with more severe disease). However, we found no correlation between QTcF prolongation and extended bedaquiline use.

Overall, our data confirm that bedaquiline should be a Group A drug in the treatment of MDR TB, as currently recommended by WHO (*6*), for not only its additive value in culture conversion and treatment success, but also its safety in varied settings.

This study has some limitations. First, cohorts differed greatly in completeness and quality of data, because they were not all initially designed for research purposes. Some variables were defined differently and the timing and number of follow-up visits varied. To maximize the use of data, we tried to work with variables that were reported across all datasets. These methodological differences, in addition to baseline differences by settings (prevalence of HIV infection, provision of baseline regimens and antiretroviral therapy), probably caused high levels of heterogeneity reflected in the meta-analyses. Second, the absence of certain variables from given datasets precluded their use in the adjusted analyses. For example, at the time of these analyses, data on pulmonary cavitation or prior use of second-line drugs were not available from the South Africa cohort, and we had end-of-treatment data for only 51.8% of those patients. Third, the absence of comparative data from patients who did not receive bedaquiline limits the inferences that can be drawn from these data. Finally, we were unable to report a causality assessment of the adverse events. Because of these limitations, the certainty of evidence was rated by the independent GDG as very low for all outcomes for the purpose of GRADE evaluation in the June 2016 meeting (*13*). Although these concerns appear to limit the credibility of these findings, they represent a real-world picture of the use of bedaquiline under programmatic conditions, outside of research settings.

The strength of this work lies in the use of patient-level data and in the random effects approach used for analyses that embraces the heterogeneity across cohorts. Our results represent data from many countries with different income levels, suggesting that the findings are generalizable. However, whereas heterogeneity is duly accounted for, it is not fully explained, and I^2^ values >50% warrant further investigation (*32*). Some study-level characteristics may contribute to the high levels of heterogeneity, such as the prevalence of HIV, extent of drug resistance, delivery of care, and data quality. Another strength of this work is the detailed information on adverse events by system, particularly the QTcF measurements, which are not usually captured in large databases and registries.

Despite the study strengths, some questions still remain unanswered, such as the role of prolonged use of bedaquiline and how best to report safety data on ECG measurements, given the heterogeneity in timing of QTcF measurements. Further studies including data on patients using bedaquiline for >6 months are warranted, as well as studies on the use of bedaquiline in shorter treatment regimens.

In conclusion, these pooled data from 5 cohorts of patients treated with bedaquiline suggest that this drug is effective and safe across different modalities of delivery and in different settings, when added to standard background regimens. Outcomes are less favorable, however, in patients with lung cavitations and more severe drug resistance. The overall certainty of the evidence is very low.

Appendix Additional information about the use of bedaquiline in treating multidrug-resistant tuberculosis.
