# Supplementary material for: Outcomes of Bedaquiline Treatment in Patients with Multidrug-Resistant Tuberculosis
Source: Emerg Infect Dis. 2019 May;25(5):936–43. doi: 10.3201/eid2505.181823 (PMC6478224; doi:10.3201/eid2505.181823)
Supplement: Appendix — Additional information about the use of bedaquiline in treating multidrug-resistant tuberculosis. [file 18-1823-Techapp-s1.pdf]

Article DOI: https://doi.org/10.3201/eid2505.181823

Outcomes of Bedaquiline Treatment in Patients with Multidrug-Resistant Tuberculosis

Appendix

Appendix Table 1. Description of samples used for the various analyses of the use of bedaquiline in treating multidrug-resistant tuberculosis

Analysis	Study cohort	Total	Description
Description of baseline characteristics	South Africa = 195<br>France = 45<br>Janssen = 205<br>Armenia = 62<br>Georgia = 30	537	Total sample with baseline characteristics available
Composition of antiretroviral therapy	South Africa = 110<br>France = 2<br>Janssen = 8	120	Data available only for HIV-infected patients on antiretroviral therapy
Effectiveness (sputum culture conversion at 6 mo)	South Africa = 72<br>France = 41<br>Janssen = 205<br>Armenia = 60<br>Georgia = 28	406	Data on patients who had a culture done at 6 mo
Treatment outcomes (cure, death, lost to follow-up, treatment complete, treatment failure)	South Africa = 101<br>France = 45<br>Janssen = 205<br>Armenia = 62<br>Georgia = 30	443	Data on cohorts of patients with complete follow-up (≥18 mo) and available outcome data
Safety (adverse events)	South Africa = 195<br>France = 45<br>Janssen = 233<br>Armenia = 62<br>Georgia = 30	565*	Total no. patients who received bedaquiline
Safety (QT prolongation)	South Africa = 141†<br>France = 45<br>Janssen = 232<br>Armenia = 62<br>Georgia = 30	510	Total number of patients with baseline and follow-up QT data

* Includes additional data from 28 patients from the Janssen cohort who received bedaquiline, but were later found to be ineligible
†Only 141 from South Africa, 1 of whom did not have baseline data

Appendix Table 2. Composition of optimized baseline regimen in studies of the use of bedaquiline in treating multidrug-resistant tuberculosis

	No. in cohort on drug (%)				
Drug	South Africa n = 195	France n = 45	Janssen n = 205	Armenia n = 62	Georgia n = 30
Aminoglycosides	56 (28.7)	45 (100.0)	152 (74.1)	17 (27.4)	1 (3.3)
Amikacin sulfate	1 (0.5)	32 (71.1)	47 (22.9)	1 (1.6)	0 (0.0)
Kanamycin	55 (28.2)	0 (0.0)	103 (50.2)	16 (25.8)	1 (3.3)
Streptomycin	0 (0.0)	45 (100.0)	3 (1.5)	0 (0.0)	0 (0.0)
Fluoroquinolones	158 (81.0)	26 (57.7)	180 (87.8)	28 (45.2)	7 (23.3)
Ciprofloxacin	0 (0.0)	0 (0.0)	7 (3.4)	0 (0.0)	0 (0.0)
Gatifloxacin	0 (0.0)	0 (0.0)	1 (0.5)	0 (0.0)	0 (0.0)
Levofloxacin	158 (81.0)	8 (17.8)	66 (32.2)	28 (45.2)	7 (23.3)
Moxifloxacin	0 (0.0)	24 (53.3)	1 (0.5)	0 (0.0)	0 (0.0)
Ofloxacin	0 (0.0)	0 (0.0)	101 (49.3)	0 (0.0)	0 (0.0)
Sparfloxacin	0 (0.0)	0 (0.0)	5 (2.4)	0 (0.0)	0 (0.0)
Macrolide	19 (9.7)	0 (0.0)	22 (10.7)	0 (0.0)	0 (0.0)
Azithromycin	9 (4.6)	0 (0.0)	16 (7.8)	0 (0.0)	0 (0.0)
Clarithromycin	10 (5.1)	0 (0.0)	6 (2.9)	0 (0.0)	0 (0.0)
Miscellaneous anti-TB drugs	170 (87.2)	45 (100.0)	205 (100.0)	62 (100.0)	30 (100.0)
Ethambutol	103 (52.8)	20 (44.4)	109 (53.2)	3 (4.8)	0 (0.0)
Isoniazid	34 (17.4)	0 (0.0)	30 (14.6)	0 (0.0)	0 (0.0)
Pyrazinamide	159 (81.5)	0 (0.0)	152 (74.1)	7 (11.3)	5 (16.7)
Rifampin	1 (0.5)	19 (42.2)	1 (0.5)	0 (0.0)	0 (0.0)
Amoxicilin clavunate	5 (2.6)	0 (0.0)	20 (9.8)	50 (80.6)	29 (96.7)
Capreomycin	38 (19.5)	3 (6.7)	45 (22.0)	22 (35.5)	11 (36.7)
Clofazimine	151 (77.4)	20 (44.4)	13 (6.3)	51 (82.3)	24 (80.0)
Cycloserine	0 (0.0)	32 (71.1)	53 (25.9)	45 (72.6)	13 (43.3)
Dapsone	0 (0.0)	0 (0.0)	1 (0.5)	0 (0.0)	0 (0.0)
Ethionamide	99 (50.8)	11 (24.4)	84 (41.0)	0 (0.0)	0 (0.0)
Imipenem	3 (1.5)	28 (62.2)	1 (0.5)	0 (0.0)	0 (0.0)
Imipenem-cilastatin	0 (0.0)	0 (0.0)	0 (0.0)	44 (71.0)	27 (90.0)
Linezolid	121 (62.1)	43 (95.6)	12 (5.9)	62 (100.0)	30 (100.0)
Para-aminosalicylic acid	157 (80.5)	40 (88.9)	97 (47.3)	31 (50.0)	12 (40.0)
Meropenem	1 (0.0)	0 (0.0)	0 (0.0)	0 (0.0)	0 (0.0)
Meropenem/amoxicilin clavunate	0 (0.0)	2 (4.4)	0 (0.0)	0 (0.0)	0 (0.0)
Para-aminosalicylic acid salt	0 (0.0)	0 (0.0)	0 (0.0)	0 (0.0)	1 (3.3)
Prothionamide	0 (0.0)	0 (0.0)	76 (37.1)	10 (16.1)	1 (3.3)
Terizidone	161 (82.6)	0 (0.0)	60 (29.3)	0 (0.0)	0 (0.0)
Thioacetazone	0 (0.0)	0 (0.0)	3 (1.5)	0 (0.0)	0 (0.0)

Appendix Table 3. Composition of regimen in patients who were on antiretroviral therapy in study of the use of bedaquiline in treating multidrug-resistant tuberculosis*

	No. in cohort on drug (%)		
Antiretroviral drug	South Africa, n = 110	France, n = 2	Janssen (n = 8)
Lamivudine	93 (47.7)	2 (4.4)	8 (100.0)
Abacavir	7 (3.6)	1 (2.2)	0 (0.0)
Zidovudine	21 (10.8)	0 (0.0)	2 (25.0)
Stavudine	27 (13.8)	0 (0.0)	0 (0.0)
Efavirenz	7 (3.6)	2 (4.4)	8 (100.0)†
Emtricitabine	15 (7.7)	1 (2.2)	0 (0.0)
Lopinavir/ritonavir	27 (14.3)	0 (0.0)	0 (0.0)
Nevirapine	74 (37.9)	0 (0.0)	1 (12.5)
Tenofovir	56 (28.7)	1 (2.2)	6 (75%)

*No patients in 2 of the 5 study cohorts were on antiretroviral therapy.
†In the Drug manufacturer cohort, EFV was used only after BDQ was stopped

Appendix Table 4. Grading of Recommendations, Assessment, Development, and Evaluation evidence profile for study of whether addition of bedaquiline to WHO-recommended second-line drug therapy safely improves outcomes for patients with multidrug-resistant tuberculosis

Certainty assessment						No. patients (weighted proportions)*	Relative effect, % at 95% CI†	Certainty	Importance
No. and design of studies	Risk for bias	Inconsistency	Indirectness	Imprecision	Other considerations				
Culture conversion (Proportion with sputum conversion at 6 mo of bedaquiline (follow up: mean 6 mo)									
5 observational studies	Serious‡	Not serious	Not serious	Not serious	None	322/405 (78.0%)	73.5–81.9	⊕◯◯◯ Very low	Critical
Mortality (follow-up: mean 18.5 mo)									
5 observational studies	Serious‡	Serious§	Not serious	Serious¶	None	49/443 (11.7%)	7.0–19.1	⊕◯◯◯ Very low	Critical
Treatment success (Proportion with treatment complete + cure) (follow-up: mean 18.5 mo)									
5 observational studies	Serious‡	Not serious	Not serious	Not serious	None	290/443 (65.8%)	59.9–71.3	⊕◯◯◯ Very low	Critical
Serious adverse events (no. patients experiencing at least one SAE over total no. patients) (follow-up: mean 18.5 mo)									
5 observational studies	Serious‡	Serious#	Serious**	Serious¶	None	47/565 (11.2%)	5.0–23.2	⊕◯◯◯ Very low	Critical
QTcF prolongation >60 ms from baseline (follow-up: mean 18.5 mo)									
5 observational studies	Serious‡	Not serious††	Not serious	Serious¶	None	75/509 (19.3%)	8.4–33.2	⊕◯◯◯ Very low	Critical
Highest recorded QTcF prolongation >500 (follow-up: mean 18.5 mo)									
5 observational studies	Serious‡	Serious‡‡	Not serious	Not serious	None	24/510 (5.8%)	1.2–13.0	⊕◯◯◯ Very low	Critical

*Patients were given bedaquiline with background MDR-TB treatment. No patients in the study were on background MBR-TB treatment (regimen drugs recommended by World Health Organization) alone.
†No comparator data were available to determine absolute effect.
‡ Downgrading for lack of control data
§Downgrading for considerable statistical heterogeneity: I^2 = 71%
¶Downgrading for wide confidence intervals
#Downgrading for considerable statistical heterogeneity: I^2 = 88%
**Downgrading for indirectness because the definition and (inconsistency in) reporting of all adverse events.
††Downgrading for statistical heterogeneity: I^2 = 93%
‡‡Downgrading for statistical heterogeneity: I^2 = 84%
